# Re-refinement of dineodymium tris­[sulfate­(VI)] tetra­hydrate

**DOI:** 10.1107/S2414314626001756

**Published:** 2026-02-27

**Authors:** Suwadee Jiajaroen, Chatphorn Theppitak, Sakchai Laksee, Kittipong Chainok

**Affiliations:** a Division of Science and Mathematic Faculty of Science and Technology Rajamangala University of Technology Tawan-ok Bangpra Sriracha Chonburi 20110 Thailand; b Chulabhorn Research Institute, Lak Si Bangkok 10210 Thailand; c Thailand Institute of Nuclear Technology (Public, Organization), Nakhon Nayok 26120, Thailand; d Thammasat University Research Unit in Multifunctional Crystalline Materials and Applications (TU-MCMA) Faculty of Science and Technology Thammasat University Khlong Luang Pathum Thani 12121 Thailand; Vienna University of Technology, Austria

**Keywords:** crystal structure, neodymium(III) sulfate, redetermination

## Abstract

The crystal structure of the title compound, poly[tetraaquatri-μ-sulfato-dineodymium], [Nd_2_(SO_4_)_3_(H_2_O)_4_]_*n*_, was re-refined from modern CCD-based single-crystal X-ray diffraction data. In comparison with the original report [Bede (1987 ▸). *Jiegou Huaxue*, **6**, 70–74], the re-refinement shows improved precision and accuracy, with all hydrogen atoms being located. The crystal structure comprises two crystallographically independent Nd^III^ sites. One adopts a capped square-anti­prismatic and the other a square-anti­prismatic coordination environment. The cations are inter­connected by bridging sulfato ligands into a framework structure that is reinforced by classical O—H⋯O hydrogen-bonding inter­actions of medium to weak strengths.

## Abstract 2

Re-refinement of [Nd_2_(SO_4_)_3_(H_2_O)_4_] led to higher precision and accuracy of the structure model, including localization of all H atoms.

## Graphical abstract

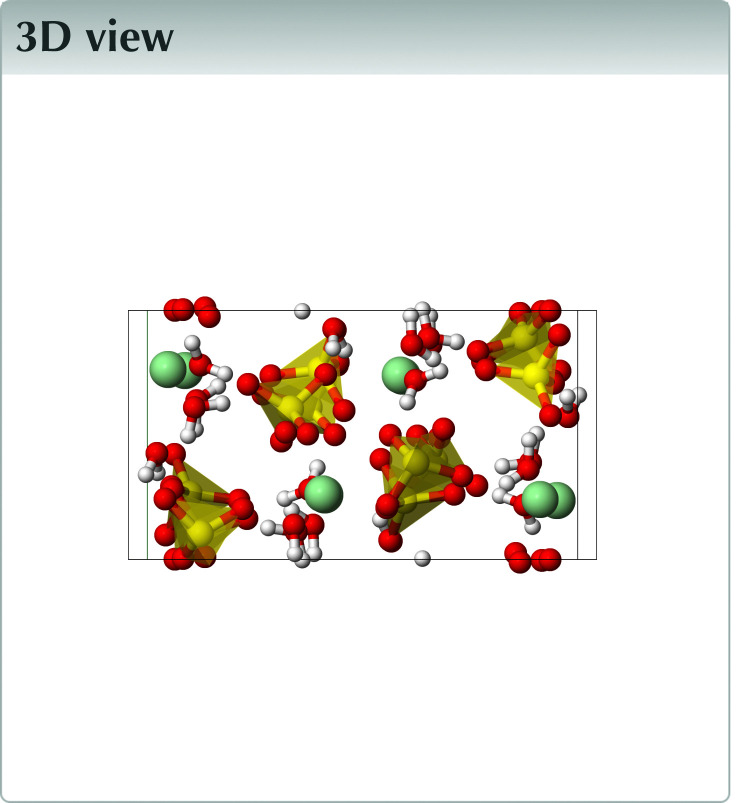

## Structure description

Coordination networks comprising lanthanide metal ions have attracted considerable inter­est in recent decades due to their structural complexity and functional properties in optical and magnetic materials, resulting from their unpaired electrons in *f*-orbitals (Patra & Pal, 2025 ▸; Mautner *et al.*, 2021 ▸; Cui *et al.*, 2012 ▸; Eliseeva & Bünzli, 2010 ▸). Since lanthanide ions favour hard donor atoms, a variety of organic ligands with oxygen atoms have been used extensively for the creation of different lanthanide coordination networks. Among them, polycarb­oxy­lic acids, particularly aromatic di­carb­oxy­lic acids like terephthalic acid (H_2_bdc) and its derivatives, have been extensively used as bridging linkers in the formation of lanthanide coordination networks (He *et al.*, 2020 ▸; Bai *et al.*, 2016 ▸), demonstrating significant luminescent properties (Alexander *et al.*, 2025 ▸). Furthermore, negatively charged polyatomic ions or oxyanions such as nitrate, sulfate, or carbonate are effective as linkers in the construction of neutral coordination networks (Yimklan *et al.*, 2024 ▸; Guo *et al.*, 2023 ▸). These networks display intriguing magnetic features attributable to the diverse coordination modes of oxyanions that connect the lanthanide cations in close proximity.

**Figure 1:**
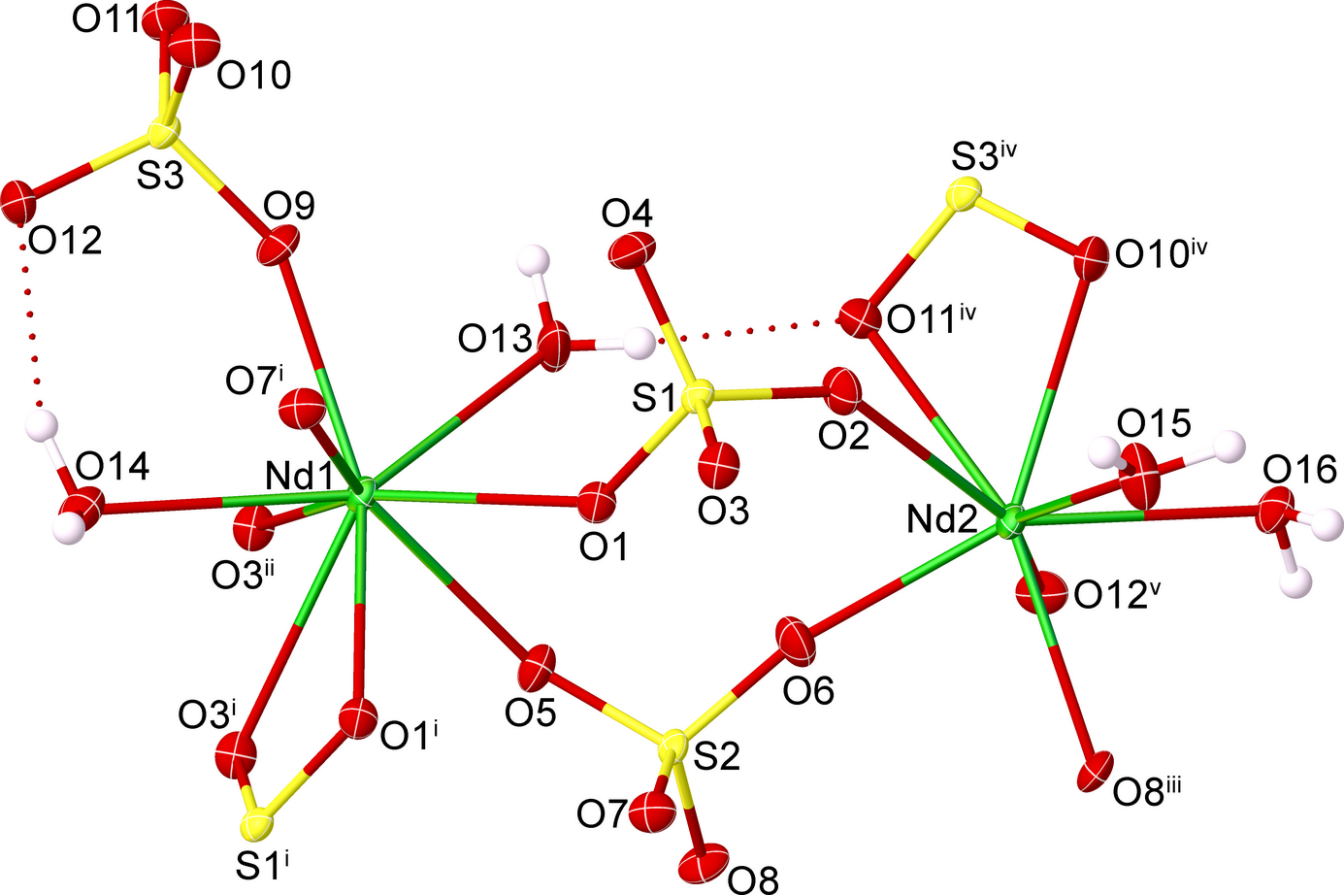
The asymmetric unit of [Nd_2_(SO_4_)_3_(H_2_O)_4_] expanded to show the full coordination spheres of the Nd atoms. Displacement ellipsoids are drawn at the 50% probability level; hydrogen-bonding inter­actions are shown as dashed lines. [Symmetry codes: (i) −*x* + 1, −*y* + 1, −*z* + 1; (ii) *x*, *y* − 1, *z*; (iii) −*x* + 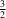, *y* + 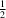, −*z* + 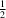; (iv) −*x* + 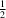, *y* + 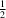, −*z* + 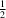; (v) *x* + 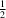, −*y* + 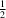, *z* − 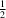.]

In the context given above, we reacted neodymium chloride with 1-(4-carb­oxy­phen­yl)-5-mercapto-1*H*-tetra­zole (cmt) under solvothermal conditions. This reaction unexpectedly yielded crystals of the inorganic title compound [Nd_2_(SO_4_)_3_(H_2_O)_4_] (Fig. 1 ▸). The sulfate anion was probably generated from the decomposition of cmt under the solvothermal conditions. A search of the Inorganic Crystal Structure Database (ICSD; Zagorac *et al.*, 2019 ▸) revealed that the crystal structure of this compound has been determined previously (collection code 68006; Bede, 1987 ▸). Isotypic lanthanide (*Ln*) crystal structures are reported for *Ln* = Ce (240937; Xu, 2008 ▸; 417417; Casari & Langer, 2007 ▸), *Ln* = Pr (422431; Kazmierczak & Hoeppe, 2011 ▸), *Ln* = La (68005; Bede, 1987 ▸), and *Ln* = Eu (420715; Choi *et al.*, 2010 ▸). The current re-refinement of [Nd_2_(SO_4_)_3_(H_2_O)_4_] provides improved precision of atomic coordinates and displacement parameters and more accurate bond lengths and bond angles. All hydrogen-atom positions were located from difference-Fourier maps and refined, allowing a more reliable description of the hydrogen-bonding inter­actions between water mol­ecules and sulfate anions (Table 1 ▸).

The asymmetric unit comprises two Nd^III^ cations (Nd1 and Nd2), three sulfate anions (S1–S3), and four coordinating water mol­ecules (O13–O16) (Fig.1). The shapes of the coordination polyhedra around the Nd^III^ cations were calculated using the *SHAPE* program (Llunell *et al.*, 2013 ▸). The Nd1 site exhibits a distorted capped square-anti­prismatic (CSAPR-9) [NdO_9_] coordination environment, comprising seven oxygen atoms from six different sulfate anions and two oxygen atoms from the coordinating water mol­ecules. The Nd2 site has a distorted square-anti­prismatic (SAPR-8) [NdO_8_] coordination environment defined by six oxygen atoms from five distinct sulfate anions and two oxygen atoms from the coordinating water mol­ecules. The Nd—O bond lengths range from 2.318 (3) to 2.676 (3) Å, while the O—Nd—O bond angles vary from 53.94 (8) to 150.36 (8)°. Bond lengths of the tetra­hedral sulfate groups are in typical ranges [1.446 (3)–1.503 (2) Å].

**Figure 2:**
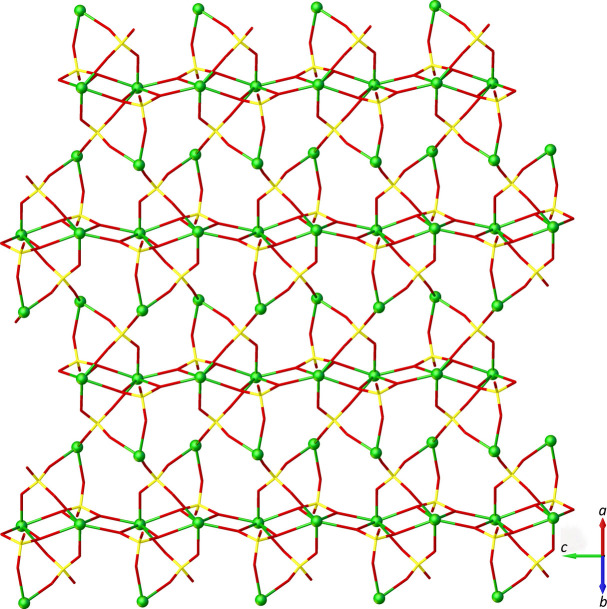
The (100) sheet in the title compound in a view along [101]. Water mol­ecules are omitted for clarity.

**Figure 3:**
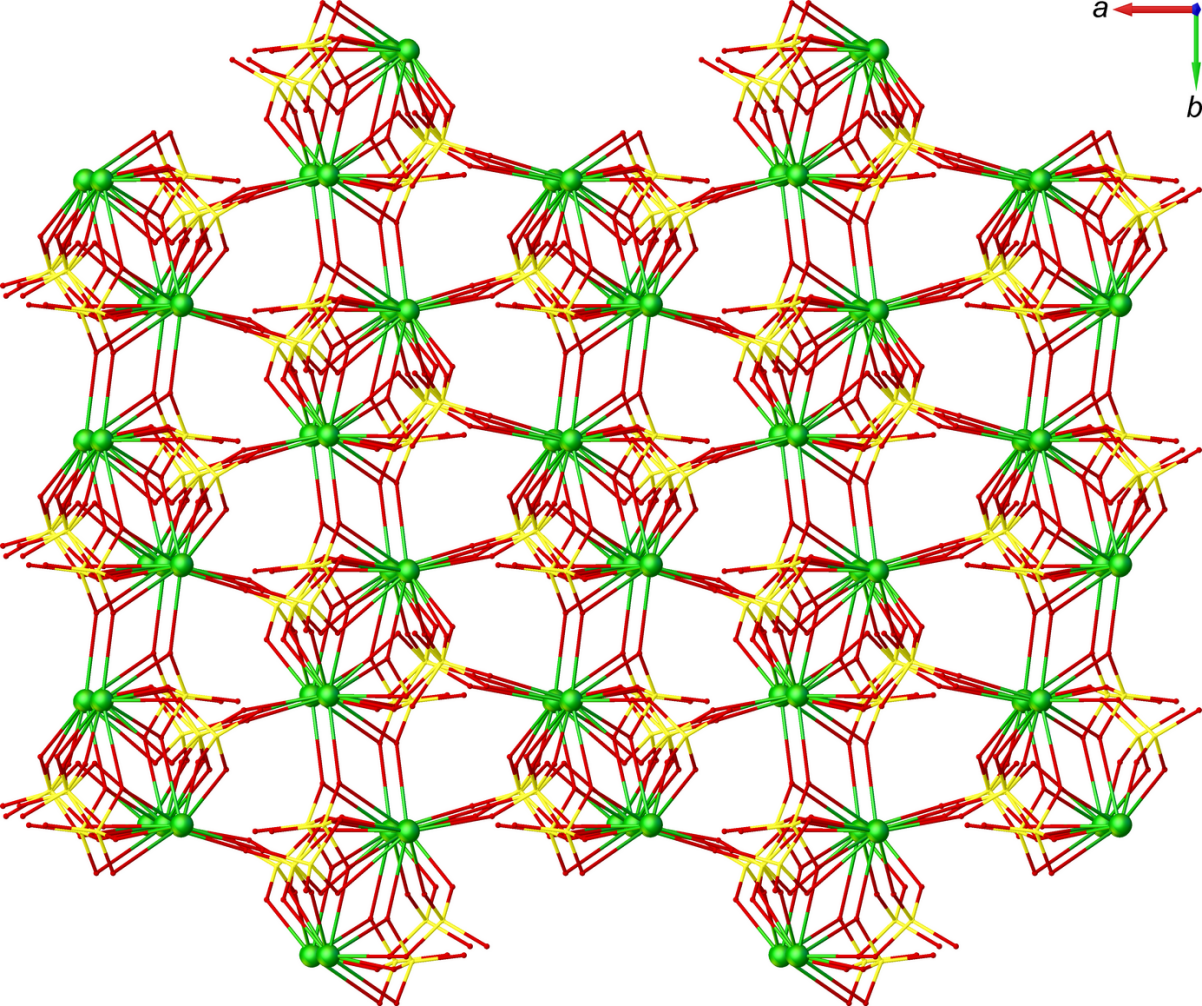
The crystal structure of the title compound in a viewe along [001].

Each Nd1 site is connected through *μ*_4_-κ^2^*O,O′*:κ*O*:κ*O′*:κ*O′′* bridging sulfato ligands, forming a double chain parallel to [010]. Nd1 and Nd2 sites are inter­linked to generate a sheet structure extending parallel (100) through *μ*_4_-κ^2^*O,O′*:κ*O*:κ*O′*:κ*O′′* and *μ*_4_-κ*O*:κ*O′*:κ*O′′*:κ*O′′′* bridging sulfato ligands (Fig. 2 ▸). These sheets are inter­connected *via* another sulfato ligand in a *μ*_3_-*κ*^2^*O*,*O*′:*κ*O:*κO’* bridging mode along [001] (Fig. 3 ▸). Hydrogen-bonding inter­actions of medium to weak strengths between water mol­ecules and sulfate O atoms, including two bifurcated hydrogen bonds (Table 1 ▸), consolidate the packing.

**Figure 4:**
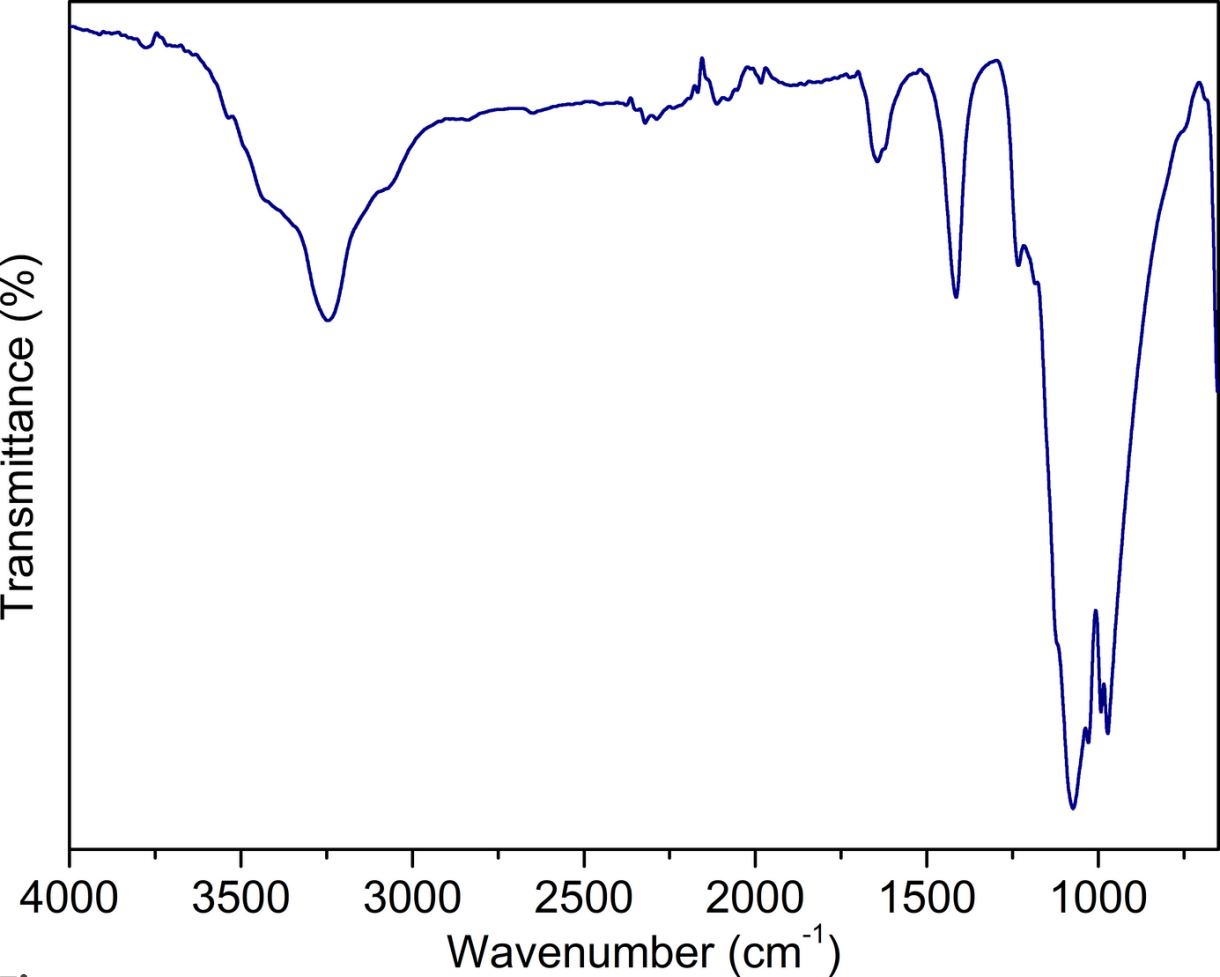
IR spectrum of the title compound.

Fig. 4 ▸ shows the infrared spectrum of the title compound. The broad absorption bands observed at around 3016 and 3502 cm^−1^ correspond to O—H stretching vibrations of coordinating water mol­ecules. The strong bands in the 972–1074 cm^−1^ region signify the vibration modes of the sulfate groups.

## Synthesis and crystallization

All reagents were of analytical grade and were used as received without further purification. A mixture solution of NdCl_3_·6H_2_O (35.9 mg, 0.1 mmol) and cmt (22.2 mg, 0.1 mmol) in mixed EtOH (3 ml) and H_2_O (2 ml) solution was added into a 15 ml Teflon lined reactor. The mixture solution was stirred at room temperature for 10 min, sealed in a stainless steel autoclave and heated in an oven at 428 K under autogenous pressure for 24 h. After cooling to room temperature and filtration, pink crystals were obtained in 45% yield (16.2 mg) based on NdCl_3_·6H_2_O.

An infrared (IR) spectrum was recorded on a Perkin-Elmer model Spectrum 100 spectrometer in the ATR mode, in the range of 650–4000 cm^−1^.

## Refinement

Crystal data, data collection and structure refinement details are summarized in Table 2 ▸.

## Supplementary Material

Crystal structure: contains datablock(s) I. DOI: 10.1107/S2414314626001756/wm5790sup1.cif

Structure factors: contains datablock(s) I. DOI: 10.1107/S2414314626001756/wm5790Isup3.hkl

CCDC reference: 2531694

Additional supporting information:  crystallographic information; 3D view; checkCIF report

## Figures and Tables

**Table 1:** Hydrogen-bond geometry (Å, °)

*D*—H⋯*A*	*D*—H	H⋯*A*	*D*⋯*A*	*D*—H⋯*A*
O13—H13*A*⋯O4^i^	0.82 (2)	2.10 (4)	2.838 (4)	149 (5)
O13—H13*B*⋯O11^ii^	0.82 (2)	2.01 (3)	2.776 (4)	156 (6)
O14—H14*A*⋯O8^iii^	0.83 (2)	2.52 (6)	3.038 (4)	121 (6)
O14—H14*A*⋯O12	0.83 (2)	2.36 (6)	2.933 (4)	127 (6)
O14—H14*B*⋯O5^iv^	0.84 (2)	1.96 (2)	2.785 (4)	166 (5)
O15—H15*A*⋯O10^v^	0.84 (2)	2.03 (3)	2.798 (4)	151 (6)
O15—H15*B*⋯O5^vi^	0.84 (2)	2.54 (6)	3.141 (4)	130 (7)
O15—H15*B*⋯O13^vi^	0.84 (2)	2.47 (5)	3.194 (4)	145 (7)
O16—H16*A*⋯O4^v^	0.82 (2)	2.02 (3)	2.799 (4)	157 (6)
O16—H16*B*⋯O10^v^	0.84 (2)	1.87 (2)	2.676 (4)	162 (5)

Symmetry codes: (i) 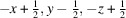; (ii) 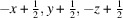; (iii) 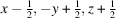; (iv) 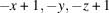; (v) 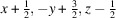; (vi) 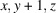.

**Table 2:** Experimental details

Crystal data	
Chemical formula	[Nd_2_(SO_4_)_3_(H_2_O)_4_]
*M*_r_	648.72
Crystal system, space group	Monoclinic, *P*2_1_/*n*
Temperature (K)	296
*a*, *b*, *c* (Å)	13.0092 (6), 7.2033 (3), 13.2968 (6)
β (°)	92.388 (2)
*V* (Å^3^)	1244.95 (10)
*Z*	4
Radiation type	Mo *K*α
μ (mm^−1^)	8.84
Crystal size (mm)	0.20 × 0.08 × 0.08

Data collection	
Diffractometer	Bruker D8 QUEST CMOS
Absorption correction	Multi-scan (*SADABS*; Krause *et al.*, 2015 ▸)
*T*_min_, *T*_max_	0.578, 0.746
No. of measured, independent and observed [*I* > 2σ(*I*)] reflections	24828, 3099, 2819
*R*_int_	0.066
(sin θ/λ)_max_ (Å^−1^)	0.668

Refinement	
*R*[*F*^2^ > 2σ(*F*^2^)], *wR*(*F*^2^), *S*	0.023, 0.059, 1.09
No. of reflections	3099
No. of parameters	223
No. of restraints	8
H-atom treatment	All H-atom parameters refined
Δρ_max_, Δρ_min_ (e Å^−3^)	1.19, −1.63

Computer programs: *APEX4* and *SAINT* (Bruker, 2021 ▸), *SHELXT* (Sheldrick, 2015*a* ▸), *SHELXL* (Sheldrick, 2015*b* ▸), *OLEX2* (Dolomanov *et al.*, 2009 ▸) and *publCIF* (Westrip, 2010 ▸).

## full crystallographic data

### Poly[tetraaquatri-µ-sulfato-dineodymium] Crystal data

**Table:**

[Nd_2_(SO_4_)_3_(H_2_O)_4_]	*F*(000) = 1216
*M**_r_* = 648.72	*D*_x_ = 3.461 Mg m^−^^3^
Monoclinic, *P*2_1_/*n*	Mo *K*α radiation, λ = 0.71073 Å
*a* = 13.0092 (6) Å	Cell parameters from 9967 reflections
*b* = 7.2033 (3) Å	θ = 3.1–28.4°
*c* = 13.2968 (6) Å	µ = 8.84 mm^−^^1^
β = 92.388 (2)°	*T* = 296 K
*V* = 1244.95 (10) Å^3^	Plate, pink
*Z* = 4	0.20 × 0.08 × 0.08 mm

### Poly[tetraaquatri-µ-sulfato-dineodymium] Data collection

**Table:**

Bruker D8 QUEST CMOS diffractometer	3099 independent reflections
Radiation source: sealed x-ray tube	2819 reflections with *I* > 2σ(*I*)
Graphite monochromator	*R*_int_ = 0.066
Detector resolution: 7.39 pixels mm^-1^	θ_max_ = 28.4°, θ_min_ = 3.1°
φ and ω scans	*h* = −17→17
Absorption correction: multi-scan (*SADABS*; Krause *et al.*, 2015)	*k* = −9→9
*T*_min_ = 0.578, *T*_max_ = 0.746	*l* = −17→17
24828 measured reflections	

### Poly[tetraaquatri-µ-sulfato-dineodymium] Refinement

**Table:**

Refinement on *F*^2^	Hydrogen site location: difference Fourier map
Least-squares matrix: full	All H-atom parameters refined
*R*[*F*^2^ > 2σ(*F*^2^)] = 0.023	*w* = 1/[σ^2^(*F*_o_^2^) + (0.0257*P*)^2^ + 2.3205*P*] where *P* = (*F*_o_^2^ + 2*F*_c_^2^)/3
*wR*(*F*^2^) = 0.059	(Δ/σ)_max_ = 0.001
*S* = 1.09	Δρ_max_ = 1.19 e Å^−^^3^
3099 reflections	Δρ_min_ = −1.62 e Å^−^^3^
223 parameters	Extinction correction: SHELXL-2019/1 (Sheldrick, 2015b), Fc^*^=kFc[1+0.001xFc^2^λ^3^/sin(2θ)]^-1/4^
8 restraints	Extinction coefficient: 0.00229 (13)

### Poly[tetraaquatri-µ-sulfato-dineodymium] Special details

**Table:**

Geometry. All esds (except the esd in the dihedral angle between two l.s. planes) are estimated using the full covariance matrix. The cell esds are taken into account individually in the estimation of esds in distances, angles and torsion angles; correlations between esds in cell parameters are only used when they are defined by crystal symmetry. An approximate (isotropic) treatment of cell esds is used for estimating esds involving l.s. planes.
Refinement. The H atoms bound to O atoms were located from difference Fourier maps and were refined with distance restraints of O—H = 0.84 ± 0.02 Å and with *U*_iso_(H) = 1.5*U*_eq_(O).

### Poly[tetraaquatri-µ-sulfato-dineodymium] Fractional atomic coordinates and isotropic or equivalent isotropic displacement parameters (Å^2^)

**Table:**

	*x*	*y*	*z*	*U*_iso_*/*U*_eq_	
Nd1	0.41471 (2)	0.25908 (2)	0.46486 (2)	0.01048 (7)	
Nd2	0.57353 (2)	0.73670 (2)	0.14948 (2)	0.01158 (7)	
S1	0.40088 (6)	0.76256 (10)	0.36613 (6)	0.01053 (17)	
S2	0.65392 (6)	0.39592 (11)	0.34841 (6)	0.01083 (16)	
S3	0.13844 (6)	0.11067 (11)	0.45661 (6)	0.01159 (16)	
O1	0.45540 (18)	0.5984 (3)	0.41346 (18)	0.0138 (5)	
O2	0.4361 (2)	0.7922 (4)	0.26405 (19)	0.0179 (5)	
O3	0.43662 (19)	0.9222 (3)	0.43036 (18)	0.0153 (5)	
O4	0.2906 (2)	0.7380 (3)	0.3657 (2)	0.0187 (6)	
O5	0.5769 (2)	0.2568 (3)	0.3787 (2)	0.0166 (6)	
O6	0.6119 (2)	0.4962 (4)	0.26066 (18)	0.0205 (5)	
O7	0.67762 (18)	0.5244 (4)	0.43216 (19)	0.0164 (5)	
O8	0.74692 (19)	0.2962 (4)	0.3207 (2)	0.0177 (5)	
O9	0.23938 (18)	0.1645 (4)	0.42137 (19)	0.0190 (5)	
O10	0.0759 (2)	0.2808 (3)	0.4724 (2)	0.0175 (5)	
O11	0.07797 (18)	0.0010 (3)	0.38166 (18)	0.0171 (5)	
O12	0.15163 (19)	0.0109 (4)	0.55274 (18)	0.0187 (5)	
O13	0.3728 (2)	0.2806 (5)	0.2807 (2)	0.0267 (7)	
H13A	0.317 (3)	0.244 (7)	0.256 (4)	0.042 (18)*	
H13B	0.389 (5)	0.369 (6)	0.246 (4)	0.062 (19)*	
O14	0.3584 (2)	0.1111 (4)	0.6308 (2)	0.0214 (6)	
H14A	0.297 (2)	0.126 (11)	0.644 (5)	0.08 (2)*	
H14B	0.372 (4)	−0.003 (3)	0.637 (4)	0.039 (14)*	
O15	0.5499 (2)	1.0738 (4)	0.1648 (2)	0.0247 (6)	
H15A	0.549 (4)	1.152 (7)	0.117 (3)	0.052 (17)*	
H15B	0.527 (6)	1.160 (8)	0.200 (5)	0.09 (3)*	
O16	0.63068 (19)	0.8621 (4)	−0.01063 (19)	0.0177 (5)	
H16A	0.677 (3)	0.805 (7)	−0.038 (4)	0.049 (17)*	
H16B	0.628 (4)	0.978 (3)	−0.018 (4)	0.032 (13)*	

### Poly[tetraaquatri-µ-sulfato-dineodymium] Atomic displacement parameters (Å^2^)

**Table:**

	*U*^11^	*U*^22^	*U*^33^	*U*^12^	*U*^13^	*U*^23^
Nd1	0.00967 (11)	0.01244 (11)	0.00959 (10)	−0.00025 (6)	0.00351 (7)	−0.00079 (6)
Nd2	0.00989 (11)	0.01386 (11)	0.01126 (11)	0.00054 (6)	0.00388 (7)	0.00217 (6)
S1	0.0091 (4)	0.0129 (4)	0.0097 (4)	0.0001 (3)	0.0029 (3)	−0.0006 (3)
S2	0.0096 (4)	0.0135 (4)	0.0097 (4)	0.0012 (3)	0.0044 (3)	0.0012 (3)
S3	0.0092 (4)	0.0148 (4)	0.0111 (4)	−0.0004 (3)	0.0030 (3)	0.0006 (3)
O1	0.0142 (11)	0.0119 (11)	0.0155 (12)	0.0019 (9)	0.0029 (9)	0.0004 (9)
O2	0.0202 (13)	0.0238 (13)	0.0101 (12)	0.0023 (11)	0.0041 (10)	0.0014 (10)
O3	0.0185 (12)	0.0128 (11)	0.0149 (12)	0.0001 (9)	0.0035 (9)	−0.0026 (9)
O4	0.0110 (13)	0.0247 (14)	0.0205 (14)	−0.0014 (9)	0.0008 (10)	0.0004 (10)
O5	0.0161 (14)	0.0160 (13)	0.0184 (13)	−0.0021 (9)	0.0069 (10)	0.0003 (9)
O6	0.0239 (13)	0.0247 (13)	0.0131 (12)	0.0039 (11)	0.0017 (10)	0.0056 (10)
O7	0.0150 (12)	0.0191 (12)	0.0151 (11)	0.0016 (10)	0.0022 (9)	−0.0039 (10)
O8	0.0106 (12)	0.0228 (12)	0.0202 (13)	0.0033 (10)	0.0055 (10)	−0.0045 (11)
O9	0.0103 (11)	0.0267 (14)	0.0204 (13)	−0.0013 (10)	0.0064 (9)	0.0019 (11)
O10	0.0174 (13)	0.0160 (12)	0.0193 (13)	0.0037 (10)	0.0021 (10)	−0.0055 (10)
O11	0.0152 (12)	0.0187 (12)	0.0172 (12)	−0.0010 (10)	−0.0008 (9)	−0.0028 (10)
O12	0.0182 (12)	0.0251 (13)	0.0128 (11)	−0.0025 (10)	0.0006 (9)	0.0058 (10)
O13	0.0252 (16)	0.0401 (17)	0.0146 (14)	−0.0146 (13)	−0.0022 (12)	0.0111 (12)
O14	0.0161 (13)	0.0179 (13)	0.0308 (15)	0.0013 (10)	0.0073 (11)	0.0047 (12)
O15	0.0394 (17)	0.0172 (13)	0.0181 (14)	0.0021 (12)	0.0067 (12)	0.0023 (11)
O16	0.0168 (12)	0.0198 (13)	0.0171 (12)	0.0001 (10)	0.0078 (10)	0.0012 (11)

### Poly[tetraaquatri-µ-sulfato-dineodymium] Geometric parameters (Å, º)

**Table:**

Nd1—O1	2.598 (2)	S1—O3	1.495 (3)
Nd1—O1^i^	2.510 (2)	S1—O4	1.446 (3)
Nd1—O3^ii^	2.489 (2)	S2—O5	1.484 (3)
Nd1—O3^i^	2.676 (3)	S2—O6	1.459 (3)
Nd1—O5	2.443 (3)	S2—O7	1.471 (3)
Nd1—O7^i^	2.427 (2)	S2—O8	1.467 (2)
Nd1—O9	2.427 (2)	S3—O9	1.465 (2)
Nd1—O13	2.491 (3)	S3—O10	1.490 (3)
Nd1—O14	2.584 (3)	S3—O11	1.473 (3)
Nd2—O2	2.430 (2)	S3—O12	1.470 (3)
Nd2—O6	2.318 (3)	O13—H13A	0.82 (2)
Nd2—O8^iii^	2.392 (2)	O13—H13B	0.82 (2)
Nd2—O10^iv^	2.499 (3)	O14—H14A	0.83 (2)
Nd2—O11^iv^	2.621 (2)	O14—H14B	0.84 (2)
Nd2—O12^v^	2.445 (2)	O15—H15A	0.84 (2)
Nd2—O15	2.457 (3)	O15—H15B	0.84 (2)
Nd2—O16	2.456 (2)	O16—H16A	0.82 (2)
S1—O1	1.503 (2)	O16—H16B	0.837 (19)
S1—O2	1.466 (3)		
			
O1^i^—Nd1—O1	69.25 (9)	O15—Nd2—O10^iv^	80.31 (9)
O1^i^—Nd1—O3^i^	53.94 (8)	O15—Nd2—O11^iv^	123.88 (9)
O1—Nd1—O3^i^	116.43 (7)	O16—Nd2—O10^iv^	69.32 (8)
O1^i^—Nd1—O14	79.94 (8)	O16—Nd2—O11^iv^	110.74 (8)
O3^ii^—Nd1—O1^i^	116.01 (8)	O16—Nd2—O15	75.62 (9)
O3^ii^—Nd1—O1	147.43 (8)	O1—S1—Nd1^i^	49.11 (10)
O3^ii^—Nd1—O3^i^	62.25 (10)	O2—S1—Nd1^i^	113.62 (11)
O3^ii^—Nd1—O13	84.36 (10)	O2—S1—O1	110.01 (14)
O3^ii^—Nd1—O14	78.11 (8)	O2—S1—O3	108.37 (15)
O5—Nd1—O1^i^	74.56 (9)	O3—S1—Nd1^i^	55.51 (10)
O5—Nd1—O1	72.16 (7)	O3—S1—O1	103.63 (15)
O5—Nd1—O3^ii^	78.41 (8)	O4—S1—Nd1^i^	134.98 (12)
O5—Nd1—O3^i^	67.75 (8)	O4—S1—O1	110.87 (14)
O5—Nd1—O13	72.38 (10)	O4—S1—O2	111.26 (17)
O5—Nd1—O14	132.77 (9)	O4—S1—O3	112.42 (15)
O7^i^—Nd1—O1	69.80 (8)	O6—S2—O5	108.43 (17)
O7^i^—Nd1—O1^i^	73.09 (8)	O6—S2—O7	110.72 (16)
O7^i^—Nd1—O3^i^	112.57 (8)	O6—S2—O8	109.10 (15)
O7^i^—Nd1—O3^ii^	142.58 (8)	O7—S2—O5	110.01 (15)
O7^i^—Nd1—O5	136.61 (8)	O8—S2—O5	108.09 (15)
O7^i^—Nd1—O9	80.36 (8)	O8—S2—O7	110.42 (15)
O7^i^—Nd1—O13	114.92 (10)	O9—S3—Nd2^vi^	123.73 (11)
O7^i^—Nd1—O14	67.57 (8)	O9—S3—O10	109.19 (16)
O9—Nd1—O1^i^	150.36 (8)	O9—S3—O11	112.71 (15)
O9—Nd1—O1	113.64 (8)	O9—S3—O12	109.51 (15)
O9—Nd1—O3^i^	129.70 (8)	O10—S3—Nd2^vi^	50.12 (11)
O9—Nd1—O3^ii^	78.24 (9)	O11—S3—Nd2^vi^	54.81 (10)
O9—Nd1—O5	135.06 (9)	O11—S3—O10	104.88 (15)
O9—Nd1—O13	67.55 (9)	O12—S3—Nd2^vi^	126.40 (10)
O9—Nd1—O14	77.92 (9)	O12—S3—O10	108.93 (16)
O13—Nd1—O1^i^	136.23 (9)	O12—S3—O11	111.46 (15)
O13—Nd1—O1	73.83 (9)	Nd1^i^—O1—Nd1	110.75 (9)
O13—Nd1—O3^i^	131.76 (9)	S1—O1—Nd1^i^	103.97 (12)
O13—Nd1—O14	143.79 (9)	S1—O1—Nd1	138.78 (14)
O14—Nd1—O1	132.89 (8)	S1—O2—Nd2	145.36 (17)
O14—Nd1—O3^i^	65.08 (8)	Nd1^vii^—O3—Nd1^i^	117.75 (10)
O2—Nd2—O10^iv^	79.18 (9)	S1—O3—Nd1^vii^	145.10 (15)
O2—Nd2—O11^iv^	68.74 (8)	S1—O3—Nd1^i^	97.07 (12)
O2—Nd2—O12^v^	141.50 (9)	S2—O5—Nd1	136.75 (14)
O2—Nd2—O15	71.81 (9)	S2—O6—Nd2	160.63 (18)
O2—Nd2—O16	137.73 (9)	S2—O7—Nd1^i^	137.62 (14)
O6—Nd2—O2	82.39 (9)	S2—O8—Nd2^viii^	148.89 (17)
O6—Nd2—O8^iii^	81.12 (9)	S3—O9—Nd1	147.58 (16)
O6—Nd2—O10^iv^	130.49 (9)	S3—O10—Nd2^vi^	102.64 (13)
O6—Nd2—O11^iv^	75.94 (9)	S3—O11—Nd2^vi^	97.84 (12)
O6—Nd2—O12^v^	72.98 (9)	S3—O12—Nd2^ix^	141.08 (16)
O6—Nd2—O15	135.23 (9)	Nd1—O13—H13A	121 (4)
O6—Nd2—O16	139.71 (9)	Nd1—O13—H13B	124 (5)
O8^iii^—Nd2—O2	127.28 (9)	H13A—O13—H13B	105 (6)
O8^iii^—Nd2—O10^iv^	144.39 (8)	Nd1—O14—H14A	116 (5)
O8^iii^—Nd2—O11^iv^	149.90 (9)	Nd1—O14—H14B	115 (3)
O8^iii^—Nd2—O12^v^	78.09 (9)	H14A—O14—H14B	108 (6)
O8^iii^—Nd2—O15	86.19 (10)	Nd2—O15—H15A	126 (4)
O8^iii^—Nd2—O16	75.46 (9)	Nd2—O15—H15B	146 (6)
O10^iv^—Nd2—O11^iv^	54.56 (8)	H15A—O15—H15B	86 (6)
O12^v^—Nd2—O10^iv^	94.64 (9)	Nd2—O16—H16A	117 (4)
O12^v^—Nd2—O11^iv^	76.73 (8)	Nd2—O16—H16B	116 (3)
O12^v^—Nd2—O15	145.15 (9)	H16A—O16—H16B	119 (5)
O12^v^—Nd2—O16	70.43 (9)		
			
Nd1^i^—S1—O1—Nd1	146.9 (2)	O5—S2—O8—Nd2^viii^	−118.4 (3)
Nd1^i^—S1—O2—Nd2	31.0 (3)	O6—S2—O5—Nd1	−75.7 (3)
Nd1^i^—S1—O3—Nd1^vii^	176.3 (3)	O6—S2—O7—Nd1^i^	60.9 (3)
Nd2^vi^—S3—O9—Nd1	141.0 (2)	O6—S2—O8—Nd2^viii^	123.9 (3)
Nd2^vi^—S3—O12—Nd2^ix^	39.3 (3)	O7—S2—O5—Nd1	45.6 (3)
O1—S1—O2—Nd2	−22.0 (3)	O7—S2—O6—Nd2	21.7 (5)
O1—S1—O3—Nd1^vii^	−173.3 (2)	O7—S2—O8—Nd2^viii^	2.0 (4)
O1—S1—O3—Nd1^i^	10.33 (13)	O8—S2—O5—Nd1	166.2 (2)
O2—S1—O1—Nd1	−108.7 (2)	O8—S2—O6—Nd2	−100.1 (5)
O2—S1—O1—Nd1^i^	104.40 (15)	O8—S2—O7—Nd1^i^	−178.1 (2)
O2—S1—O3—Nd1^vii^	69.8 (3)	O9—S3—O10—Nd2^vi^	118.38 (14)
O2—S1—O3—Nd1^i^	−106.50 (13)	O9—S3—O11—Nd2^vi^	−116.20 (14)
O3—S1—O1—Nd1	135.65 (19)	O9—S3—O12—Nd2^ix^	−147.5 (2)
O3—S1—O1—Nd1^i^	−11.28 (14)	O10—S3—O9—Nd1	86.7 (3)
O3—S1—O2—Nd2	90.6 (3)	O10—S3—O11—Nd2^vi^	2.48 (15)
O4—S1—O1—Nd1^i^	−132.11 (14)	O10—S3—O12—Nd2^ix^	93.1 (3)
O4—S1—O1—Nd1	14.8 (3)	O11—S3—O9—Nd1	−157.1 (3)
O4—S1—O2—Nd2	−145.3 (3)	O11—S3—O10—Nd2^vi^	−2.64 (16)
O4—S1—O3—Nd1^i^	130.11 (13)	O11—S3—O12—Nd2^ix^	−22.1 (3)
O4—S1—O3—Nd1^vii^	−53.6 (3)	O12—S3—O9—Nd1	−32.5 (4)
O5—S2—O6—Nd2	142.4 (5)	O12—S3—O10—Nd2^vi^	−122.07 (14)
O5—S2—O7—Nd1^i^	−58.9 (3)	O12—S3—O11—Nd2^vi^	120.19 (13)

Symmetry codes: (i) −*x*+1, −*y*+1, −*z*+1; (ii) *x*, *y*−1, *z*; (iii) −*x*+3/2, *y*+1/2, −*z*+1/2; (iv) −*x*+1/2, *y*+1/2, −*z*+1/2; (v) *x*+1/2, −*y*+1/2, *z*−1/2; (vi) −*x*+1/2, *y*−1/2, −*z*+1/2; (vii) *x*, *y*+1, *z*; (viii) −*x*+3/2, *y*−1/2, −*z*+1/2; (ix) *x*−1/2, −*y*+1/2, *z*+1/2.

### Poly[tetraaquatri-µ-sulfato-dineodymium] Hydrogen-bond geometry (Å, º)

**Table:**

*D*—H···*A*	*D*—H	H···*A*	*D*···*A*	*D*—H···*A*
O13—H13*A*···O4^vi^	0.82 (2)	2.10 (4)	2.838 (4)	149 (5)
O13—H13*B*···O11^iv^	0.82 (2)	2.01 (3)	2.776 (4)	156 (6)
O14—H14*A*···O8^ix^	0.83 (2)	2.52 (6)	3.038 (4)	121 (6)
O14—H14*A*···O12	0.83 (2)	2.36 (6)	2.933 (4)	127 (6)
O14—H14*B*···O5^x^	0.84 (2)	1.96 (2)	2.785 (4)	166 (5)
O15—H15*A*···O10^xi^	0.84 (2)	2.03 (3)	2.798 (4)	151 (6)
O15—H15*B*···O5^vii^	0.84 (2)	2.54 (6)	3.141 (4)	130 (7)
O15—H15*B*···O13^vii^	0.84 (2)	2.47 (5)	3.194 (4)	145 (7)
O16—H16*A*···O4^xi^	0.82 (2)	2.02 (3)	2.799 (4)	157 (6)
O16—H16*B*···O10^xi^	0.84 (2)	1.87 (2)	2.676 (4)	162 (5)

Symmetry codes: (iv) −*x*+1/2, *y*+1/2, −*z*+1/2; (vi) −*x*+1/2, *y*−1/2, −*z*+1/2; (vii) *x*, *y*+1, *z*; (ix) *x*−1/2, −*y*+1/2, *z*+1/2; (x) −*x*+1, −*y*, −*z*+1; (xi) *x*+1/2, −*y*+3/2, *z*−1/2.
